# Benchmarking the diagnostic performance of open source LLMs in 1933 Eurorad case reports

**DOI:** 10.1038/s41746-025-01488-3

**Published:** 2025-02-12

**Authors:** Su Hwan Kim, Severin Schramm, Lisa C. Adams, Rickmer Braren, Keno K. Bressem, Matthias Keicher, Paul-Sören Platzek, Karolin Johanna Paprottka, Claus Zimmer, Dennis M. Hedderich, Benedikt Wiestler

**Affiliations:** 1 Department of Diagnostic and Interventional Neuroradiology, Klinikum rechts der Isar, School of Medicine and Health, Technical University of Munich, Munich, Germany; 2 Department of Diagnostic and Interventional Radiology, Klinikum rechts der Isar, School of Medicine and Health, Technical University of Munich, Munich, Germany; 3 Department of Cardiovascular Radiology and Nuclear Medicine, German Heart Center Munich, School of Medicine and Health, Technical University of Munich, Munich, Germany; 4 Computer Aided Medical Procedures, Technical University of Munich, Munich, Germany; 5 AI for Image-Guided Diagnosis and Therapy, School of Medicine and Health, Technical University of Munich, Munich, Germany

**Keywords:** Diagnosis, Medical imaging, Translational research

## Abstract

Recent advancements in large language models (LLMs) have created new ways to support radiological diagnostics. While both open-source and proprietary LLMs can address privacy concerns through local or cloud deployment, open-source models provide advantages in continuity of access, and potentially lower costs. This study evaluated the diagnostic performance of fifteen open-source LLMs and one closed-source LLM (GPT-4o) in 1,933 cases from the Eurorad library. LLMs provided differential diagnoses based on clinical history and imaging findings. Responses were considered correct if the true diagnosis appeared in the top three suggestions. Models were further tested on 60 non-public brain MRI cases from a tertiary hospital to assess generalizability. In both datasets, GPT-4o demonstrated superior performance, closely followed by Llama-3-70B, revealing how open-source LLMs are rapidly closing the gap to proprietary models. Our findings highlight the potential of open-source LLMs as decision support tools for radiological differential diagnosis in challenging, real-world cases.

## Introduction

Recent advancements in artificial intelligence (AI) have transformed medical diagnostics, offering innovative tools to support clinical decision-making. One promising development is the emergence of large language models (LLMs), which excel at processing and generating natural language. In radiology, these models have demonstrated potential in various applications, including defining study protocols^1,2^, performing differential diagnosis^3,4^, generating reports^5,6^, and extracting information from free-text reports^7,8^.

However, a significant barrier to widespread clinical adoption is data privacy. The LLMs primarily used in previous studies are proprietary, closed-source models, such as GPT-4, Claude 3, or Gemini^9–11^. Access to these models is typically provided via web-based interfaces or via application programming interfaces (API), both of which necessitate the transfer of data to third-party servers, thereby increasing the risk of unauthorized access or misuse of sensitive health information and limiting their use on patient data. While cloud-based solutions for proprietary LLMs can address some privacy concerns, they may still be subject to commercial update cycles and potentially higher long-term costs.

Open-source models offer a viable alternative, enabling care institutions to retain patient data within their local infrastructure, mitigating these privacy concerns, and providing continuity of access independent of commercial update cycles, which can lower costs due to their free availability. While historically open-source LLMs have underperformed in clinical decision support tasks^12,13^, Meta’s latest Llama-3 has shown performance levels on par with leading proprietary models in some areas, such as answering radiology board exam questions^14^. However, the diagnostic accuracy of such models in real-world clinical cases remains largely unexplored.

A well-suited resource for such an evaluation is Eurorad, a comprehensive repository of peer-reviewed radiological case reports managed by the European Society of Radiology (ESR). Eurorad serves as a valuable educational resource for radiologists, residents, and medical students, and encompasses a wide range of cases across radiological subspecialties such as abdominal imaging, neuroradiology, uroradiology, and pediatric radiology^15^.

Therefore, the aim of this study was to evaluate the performance of state-of-the-art open-source LLMs in radiological diagnosis using Eurorad case reports.

## Results

### Dataset

The initial dataset retrieved from the Eurorad library consisted of 4827 case reports. Using the Llama-3-70B model, we identified 2894 cases where the diagnosis was explicitly stated within the case description. These cases were subsequently excluded, resulting in a final dataset of 1933 cases for analysis. This filtering process ensured that the LLMs were evaluated on genuinely challenging cases that required inference rather than simple information extraction. The dataset was primarily composed of cases from neuroradiology (21.4%), abdominal imaging (18.1%), and musculoskeletal imaging (14.6%), whereas breast imaging (3.4%) and interventional radiology (1.4%) were underrepresented (Table 1). This distribution broadly reflects the relative prevalence of different radiological subspecialties in clinical practice.

**Table 1:** Dataset composition by subspecialty

Subspecialty	No. cases	Proportion
Neuroradiology	413	21.4%
Abdominal imaging	349	18.1%
Musculoskeletal system	282	14.6%
Chest imaging	190	9.8%
Uroradiology & genital male imaging	151	7.8%
Pediatric radiology	137	7.1%
Head & neck imaging	124	6.4%
Cardiovascular	112	5.8%
Genital (female) imaging	83	4.3%
Breast imaging	65	3.4%
Interventional radiology	27	1.4%
Total	1933	100.0%

### LLM judge performance

To use Llama-3-70B as an automated LLM Judge for assessing model responses in the large Eurorad dataset, we first needed to calibrate its response assessment against human expert assessment. In a subset of 140 Eurorad cases, Llama-3-70B exhibited a high accuracy of 87.8% in classifying responses as “correct” or “incorrect” (123 out of 140 responses; 95% CI: 0.82–0.93). Furthermore, in a separate subset of 20 responses that were rated by all three radiologists, the interrater agreement was found to be 100%, indicating complete consensus among the human experts.

The high level of agreement between Llama-3-70B and the human radiologists, as well as the complete consensus among the radiologists themselves, supports the validity of using Llama-3-70B as an automated judge for the larger LLM response dataset. This allows us to include the small inaccuracies of Llama-3-70B in the overall confidence interval assessment, as detailed in the “Statistics” section of the Methods.

### Model performance

Across all models, the highest levels of diagnostic accuracy were achieved in interventional radiology (67.8 ± 6.2%), cardiovascular imaging (62.5 ± 3.2%), and abdominal imaging (60.5 ± 1.8%), whereas lower accuracy was observed in breast imaging (50.0 ± 4.3%) and musculoskeletal imaging (50.4 ± 2.1%). Granular accuracy metrics by subspecialty and model are provided in Supplementary Table 2.

**Fig. 1:**
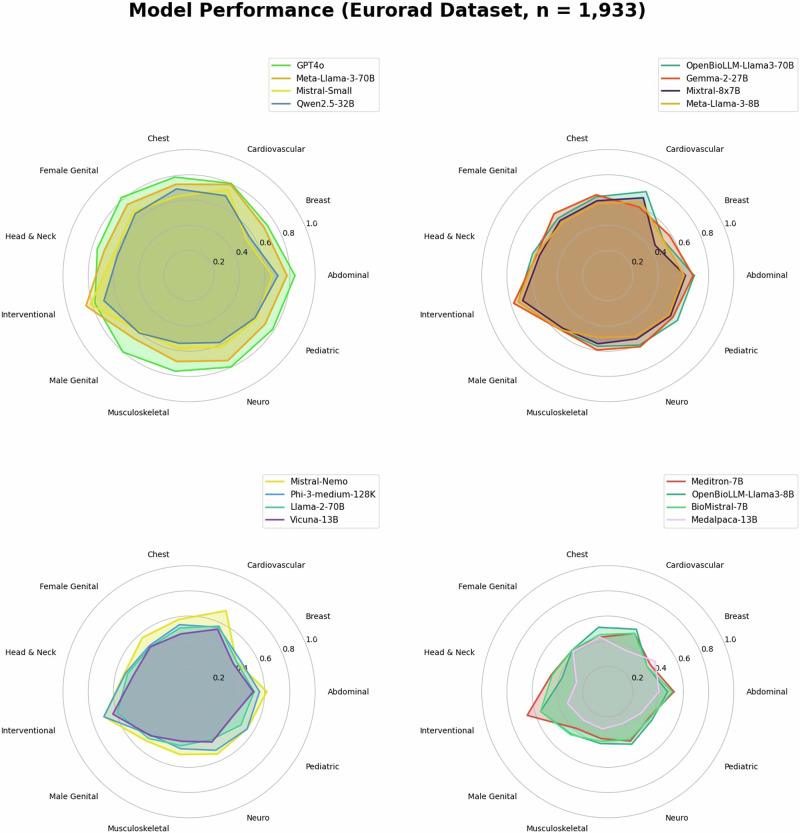
Model performance across subspecialty. Models were ranked by overall accuracy and grouped into radar plots, with four models displayed per plot. The four top-performing models are shown in the top left corner.

**Fig. 2:**
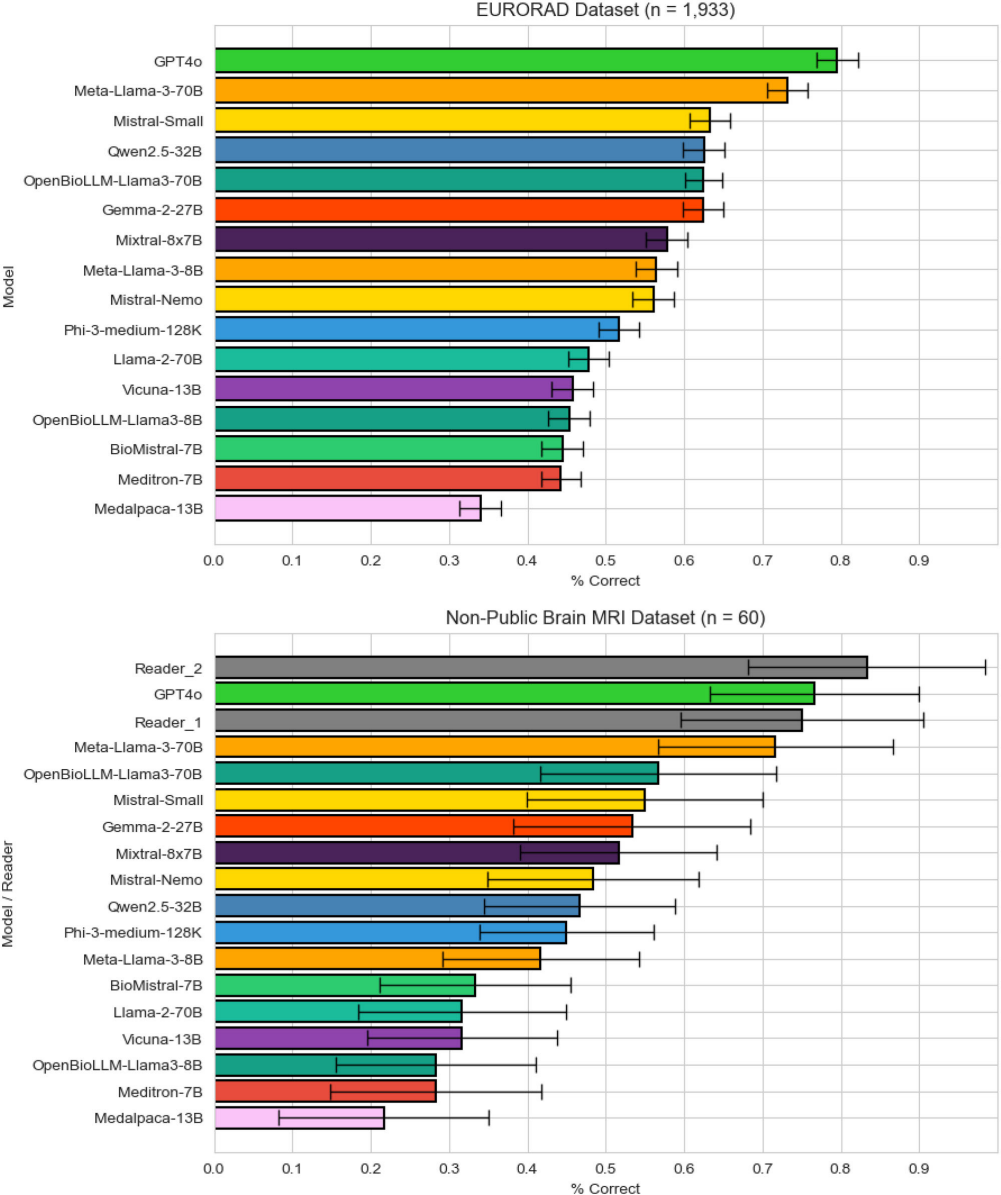
Performance of open-source LLMs in Eurorad dataset (*n* = 1933) and local brain MRI dataset (*n* = 60). Error bars indicate adjusted 95% confidence intervals. Reader 1 and 2 were radiologists with two and four years of dedicated neuroradiology experience each.

GPT-4o demonstrated superior diagnostic performance across all subspecialties except interventional radiology, achieving a rate of 79.6 ± 2.3% correct responses. Meta-Llama-3-70B revealed the highest performance among open-source LLMs (73.2 ± 2.5%), with a considerable margin ahead of Mistral-Small (63.3 ± 2.6%), Qwen2.5-32B (62.5 ± 2.6%), and OpenBioLLM-Llama-3-70B (62.5 ± 2.6%). Lowest performance was seen in Medalpaca-13B (34.0 ± 2.6%), Meditron-7B (44.3 ± 2.7%), and BioMistral-7B (44.5 ± 2.7%). Meta-Llama-3-70B’s accuracy was substantially higher than its predecessor model Meta-Llama-2-70B (Figs. 1, 2).

In the local brain MRI dataset, similar results were observed, with GPT-4o (76.7 ± 15.1%) and Llama-3-70B (71.7 ± 12.2%) again leading the rankings. Reader 2, a board-certified neuroradiologist, achieved the highest accuracy with 83.3 ± 13.3% correct responses. Reader 1, a radiologist with 2 years of neuroradiology experience achieved rates comparable to GPT-4o and Meta-Llama-3-70B (75.0 ± 15.5%). Several other models showed a drop in performance levels in the local dataset of up to 16% (e.g., Llama-2-70B: 47.8 ± 2.7% to 31.7 ± 12.6%) (Fig. 2).

### Correlation analysis

**Fig. 3:**
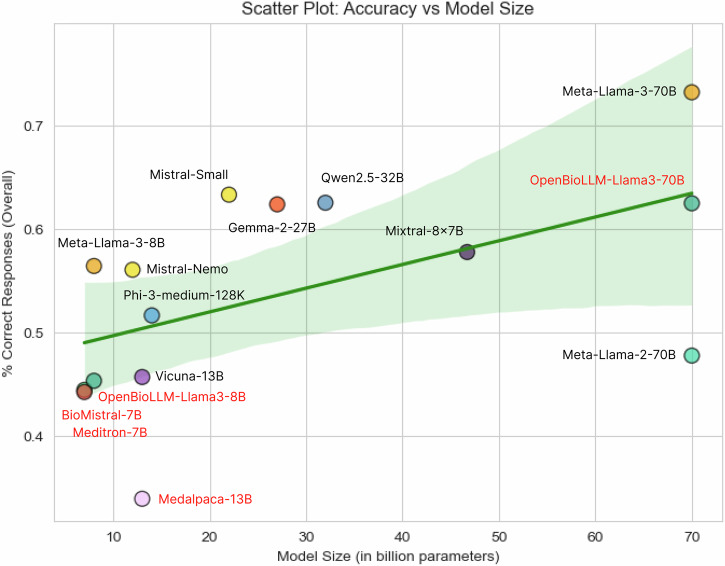
Scatter plot: accuracy vs model size. Models fine-tuned with biomedical corpora are highlighted in red. A Pearson correlation coefficient of 0.54 was determined, indicating a moderate positive correlation.

The relationship between model accuracy and model size (in billion parameters) is illustrated in Fig. 3. A Pearson correlation coefficient of 0.54 was determined, indicating a moderate positive correlation.

LLMs fine-tuned with domain-specific training data showed lower accuracy compared to general-purpose models of comparable size. For instance, both OpenBioLLM-Llama-3-70B (62.4 ± 2.6%) and OpenBioLLM-Llama-3-8B (45.4 ± 2.7%) demonstrated performance levels inferior to their respective base models Meta-Llama-3-70B (73.2 ± 2.5%) and Meta-Llama-3-8B (56.4 ± 2.6%).

## Discussion

In this study, we benchmarked the diagnostic performance of fifteen leading open-source LLMs in a heterogeneous, challenging cohort of 1933 peer-reviewed case reports from the Eurorad library. Although GPT-4o outperformed all included open-source LLMs (79.6%), Meta’s Llama-3-70B followed very closely (73.2%), highlighting how open-source LLMS are quickly closing the gap to proprietary LLMs. This level of performance is noteworthy given the complexity and diversity of the cases included in our dataset. In the local brain MRI dataset, both models reached accuracy rates comparable to or only slightly lower than those of two experienced radiologists. The remaining models followed with a sizeable margin, underscoring the current dominance of Llama-3 among open-source models. This trend is further supported by Llama-3’s proficiency in other clinical tasks, such as answering close-ended medical questions, summarizing clinical documents, and patient education^14,16^.

Importantly, this study assessed the diagnostic performance of LLMs based on real case descriptions, more accurately representing the complexities of real-life clinical decision-making than questions with pre-defined response options. This approach provides a more realistic evaluation of LLMs’ potential in clinical settings, where the ability to interpret nuanced clinical information is crucial.

Our results revealed variations in performance across radiological subspecialties, with higher accuracy in genital (female) imaging and lower accuracy in musculoskeletal imaging. These differences may reflect inherent complexities within each subspecialty, variations in the quality or specificity of case descriptions, or potential biases in the models’ training data. Further investigation into these subspecialty-specific performance variations could provide valuable insights for targeted model improvements and clinical applications.

Although we observed a moderate positive correlation between model size and diagnostic accuracy, some lighter models such as Meta-Llama-3-8B exhibited strong performance, outperforming larger models with more parameters (e.g., Llama-2-70B and Vicuna-13B). This suggests that smaller, lower-cost models with nonetheless robust results are attainable, making the implementation of LLMs in resource-constrained healthcare settings more viable. Interestingly, medically fine-tuned models tended to perform worse than their respective base model or other general-purpose models of comparable size. This finding challenges the widely held assumption that domain-adaptive pretraining enhances model performance, although some recent studies support our observation^17,18^.

Employing a state-of-the-art LLM model to automate the evaluation of LLM responses facilitated the large-scale analysis of thousands of cases, a scope unrealizable through manual processing. This strategy establishes a methodical benchmark for future large-scale investigations of clinical text documents.

Overall, this study highlights the potential of open-source LLMs as decision-support tools for radiological differential diagnosis in real-world cases. Yet, several obstacles for successful implementation and adoption remain to be addressed.

To begin with, how physicians can effectively interact with LLMs and how potential risks can be mitigated is yet to be determined. Whereas this study investigated the isolated diagnostic performance of LLMs, it is more realistic for them to serve as tools enhancing the capabilities of physicians^19,20^. In the context of radiological diagnosis, LLMs could help rapidly generate multiple hypotheses that require further validation. A potential threat originating from LLM suggestions is automation bias, which is the common tendency of humans to excessively rely on automated decision-making systems. This cognitive phenomenon has been observed in AI-based systems for mammography classification^21^ and cerebral aneurysm detection^22^, and could lead to systematic errors in physicians who fail to critically evaluate LLM suggestions. In contrast, LLMs could also play a role in reducing cognitive biases if they are intentionally utilized to provide different perspectives and uncover common fallacies^23^.

While effective human-AI interaction is crucial, practical implementation of LLMs hinges on overcoming technical barriers. Operating open-source LLMs locally requires an adequate hardware and software infrastructure, as well as IT expertise that might be available in large academic centers but not in smaller institutions or practices^24^. Vendors of PACS, RIS, or EHR systems could be instrumental in overcoming these barriers by integrating LLM-based features in a privacy-preserving and user-friendly manner.

In addition to technical considerations, economic implications from a healthcare provider and system perspective warrant careful examination. Deploying LLMs incurs costs related to infrastructure, query usage, and physician training. Cost-effectiveness studies are needed to assess whether these investments are justified by the potential gains in diagnostic accuracy, productivity, and patient outcomes.

Moreover, establishing effective regulatory frameworks for the development and use of LLM-based tools in medicine remains a considerable challenge. LLMs used in clinical decision-making should meet rigorous safety and reliability standards, yet the near-infinite range of inputs and outputs complicates the definition of comprehensive guidelines^25^. Regulatory authorities such as the FDA (Food and Drug Administration) and the EMA (European Medicines Agency) should aim for adaptable yet robust oversight mechanisms to harness the potential benefits while preventing potential patient harm.

Lastly, patient perception of AI-enhanced diagnosis is another critical factor to consider. Transparent communication about the role of LLMs in the diagnostic process will likely ensuring patient trust, ultimately fostering acceptance^26^.

This study has several limitations. First, data contamination of LLMs cannot be definitively ruled out. Given the lack of transparency regarding the LLM training datasets, it is possible that the case reports used in this study overlap with the training data of some models. However, our complementary assessment on a non-public brain MRI dataset revealed largely consistent overall model rankings, although some models exhibited diminished performance. The detection and estimation of data contamination is an active area of research, and several methods have been proposed^27–30^. In one approach, the LLM in question is instructed to complete the initial segment of a dataset partition, and the overlap of the LLM output with the original data is measured^27^. Another framework evaluates data contamination based on LLM output distribution characteristics^30^. Despite progress, significant weaknesses such as limited reproducibility and the lack of baseline comparisons persist, and robust, validated methods for detecting data contamination are yet to be established^29^.

Second, while the use of an LLM for the evaluation of LLM responses significantly enhanced the scalability of the analysis, it did so at the expense of reduced accuracy. To mitigate this limitation, we adjusted the standard error of model performance assessment based on our evaluation of Llama-3-70B’s judging accuracy in a subset of the data.

Third, this study did not evaluate the multimodal performance of vision-language models (VLMs) capable of ingesting both text and image data as input. Both closed-source (e.g., GPT-4o, Claude 3 Opus, Gemini 1.5 Pro) and open-source VLMs (e.g., LLaVA-1.5, Qwen-VL, CogVLM)^31–36^ have become available, and their role in radiology report generation (either standalone^37^ or as an aid to radiologists^38^) is being explored. Several studies further evaluated the performance of VLMs in differential diagnosis but showed mixed results^4,10,39,40^. Whereas most VLMs are not capable of processing 3-dimensional image inputs, new models supporting 3D CT or MRI scans have been developed^41,42^.

Fourth, we did not investigate the impact of temperature settings or prompt design on LLM performance. To ensure deterministic responses, we applied a temperature of 0, but higher temperatures could potentially improve diagnostic accuracy^10^. Similarly, the optimal task-specific prompting strategy for radiological diagnosis is yet to be determined^43^.

Finally, this study did not account for the influence of varying descriptions of the same case.

A recent study evaluating GPT-4(V) in radiological diagnosis revealed that image description is a major determinant of LLM accuracy^4^. The Eurorad case descriptions were written in awareness of the correct diagnosis, and their use of specific terminology or emphasis on certain image characteristics might have introduced a positive bias in LLM performance.

In conclusion, we found that several open-source LLMs demonstrate promising performance in identifying the correct diagnosis based on case descriptions from the Eurorad library, highlighting their potential as a decision-support tool for radiological differential diagnosis.

## Methods

The need for informed consent was waived by the Ethics Committee of the Technical University of Munich, as it involved the retrospective analysis of publicly available data and de-identified local data, posing minimal risk to the participants.

### Data

To create a comprehensive and diverse dataset of challenging radiology cases, we automatically downloaded case report data—including “Clinical History,” “Imaging Findings,” “Final Diagnosis,” and “Section”—from the European Society of Radiology’s case report library at https://eurorad.org/. Containing information on patient demographics, symptoms, pre-existing conditions, lab values, and detailed imaging findings, the case descriptions were sufficient to determine the accurate diagnosis in most cases. The final diagnosis, as indicated in the Eurorad dataset, served as the ground truth.

All case reports published after July 6, 2015, and licensed under the Creative Commons License CC BY-NC-SA 4.0, were scraped using the Python library “Scrapy” (version 2.11.2) on June 15, 2024.

To address potential data contamination concerns and assess generalizability, we further validated the performance of LLMs in a local dataset of 60 brain MRI cases. These were obtained from our local imaging database, as reported previously^4^, and equally contained a brief clinical history and imaging findings. True diagnoses in the local dataset were determined based either on histopathology or through the independent agreement of at least two neuroradiologists, taking into account all relevant clinical follow-up information. This local dataset is not publicly accessible and, thus, highly unlikely to have been included in the LLMs’ training data.

### LLM selection

GPT-4o was included as a state-of-the-art closed-source LLM by OpenAI. Among open-source LLMs, general-purpose models from leading developers (Meta, Microsoft, Mistral, Alibaba, and Google) and top-ranking medically fine-tuned LLMs chosen based on trend and download metrics on HuggingFace (https://huggingface.co/models) were selected. One medically fine-tuned model, Meditron-70B, was tested but eventually excluded from the analysis as it returned non-sensical responses, possibly because it was not specifically trained to execute user instructions (also known as “instruction fine-tuning”).

### LLM setup

To evaluate a range of open-source large language models (LLMs), we developed a Python-based workflow utilizing the “llama_cpp_python” library (version 0.2.79). This library provides Python bindings for the widely-used “llama_cpp” software, enabling the execution of local, quantized LLMs in GGUF (GPT-generated unified format). Quantization involves reducing the precision of the model’s numerical weights, typically transitioning from floating-point to lower-bit representations, which results in a smaller and faster model while preserving performance. For most models, Q5_K_M was chosen as a quantization, typically offering a good balance between compression and quality. For the 70B models, a quantization factor of Q4_K_M was selected to allow full GPU offloading. The “llama_cpp_python” library allows for detailed control over relevant hyperparameters. In our experiments, we fully offloaded the LLMs to a GPU for higher computational speed, set the temperature to 0 to ensure deterministic responses, and limited the context width to 1024 tokens, which we previously validated to accommodate all case reports and responses. We chose these settings to balance performance and reproducibility, although we acknowledge that different configurations might yield varying results. Our Python code for prompt construction, along with detailed links to all models (downloaded from https://huggingface.co/), is publicly available in our GitHub repository at https://github.com/ai-idt/os_llm_eurorad. The fifteen open-source LLM models included in this study are detailed in Table 2. All experiments were conducted using an Nvidia P8000 GPU with 48GB of video memory.

**Table 2:** Open-source LLM details

Model	# Params	Base model	HuggingFace Link	Reference
BioMistral-7B	7B	Mistral-7B	https://huggingface.co/BioMistral/BioMistral-7B-DARE-GGUF	https://arxiv.org/abs/2402.10373
Gemma-2-27B	27B	Gemma-2	https://huggingface.co/bartowski/gemma-2-27b-it-GGUF	https://arxiv.org/abs/2408.00118
Llama-2-70B	70B	Llama-2	https://huggingface.co/TheBloke/Llama-2-70B-Chat-GGUF	https://arxiv.org/abs/2307.09288
Medalpaca-13B	13B	LLaMA	https://huggingface.co/mradermacher/medalpaca-13b-GGUF	https://arxiv.org/abs/2304.08247
Meditron-7B	7B	Llama-2	https://huggingface.co/TheBloke/meditron-7B-chat-GGUF	https://arxiv.org/abs/2311.16079
Meta-Llama-3-8B	8B	Llama-3	https://huggingface.co/bartowski/Meta-Llama-3-8B-Instruct-GGUF	https://arxiv.org/abs/2407.21783
Meta-Llama-3-70B	70B	Llama-3	https://huggingface.co/bartowski/Meta-Llama-3-70B-Instruct-GGUF	https://arxiv.org/abs/2407.21783
Mistral-Nemo	12B	Mistral-Nemo	https://huggingface.co/bartowski/Mistral-Nemo-Instruct-2407-GGUF	https://mistral.ai/news/mistral-nemo/
Mistral-Small	22B	Mistral-Small	https://huggingface.co/bartowski/Mistral-Small-Instruct-2409-GGUF	https://mistral.ai/news/september-24-release/
Mixtral-8x7B	46.7B	Mistral-7B	https://huggingface.co/TheBloke/Mixtral-8x7B-Instruct-v0.1-GGUF	https://arxiv.org/abs/2401.04088
OpenBioLLM-Llama-3-8B	8B	Llama-3	https://huggingface.co/bartowski/OpenBioLLM-Llama3-8B-GGUF	https://huggingface.co/blog/aaditya/openbiollm
OpenBioLLM-Llama3-70B	70B	Llama-3	https://huggingface.co/mradermacher/OpenBioLLM-Llama3-70B-GGUF	https://huggingface.co/blog/aaditya/openbiollm
Phi-3-medium-128K	14B	Phi-3	https://huggingface.co/bartowski/Phi-3-medium-128k-instruct-GGUF	https://arxiv.org/abs/2404.14219
Qwen2.5-32B	32B	Qwen-2.5	https://huggingface.co/Qwen/Qwen2.5-32B-Instruct-GGUF	https://qwenlm.github.io/blog/qwen2.5/ & https://arxiv.org/abs/2407.10671
Vicuna-13B	13B	Llama-2	https://huggingface.co/TheBloke/vicuna-13B-v1.5-GGUF	https://lmsys.org/blog/2023-03-30-vicuna/

GPT-4o (“gpt-4o-2024-08-06”) was accessed via OpenAI’s application programming interface (API) (https://platform.openai.com/docs/models#gpt-4o).

### Human reader performance

To contrast the diagnostic performance of LLMs with radiologists, two readers were instructed to provide up to three differential diagnoses for the local dataset of 60 brain MRI cases. Reader 1 was a radiologist with two years of dedicated neuroradiology experience, while Reader 2 was a board-certified neuroradiologist with four years of experience. To create equal conditions, the two readers were provided with the textual case descriptions but not the image data, consistent with the setup for the LLMs, even though this did not represent a realistic clinical scenario.

### Case selection and response assessment

Upon review, we noted that a significant proportion of cases already contained the correct diagnosis within the “Clinical History” and “Imaging Findings” sections. Drawing inspiration from the “LLM-as-a-Judge” paradigm^44^, we employed the most advanced open-source model available at the outset of this study, Llama-3-70B, to filter out these cases. A recent study indicated that Llama-3-70B, along with GPT-4 Turbo, demonstrated the closest alignment with human evaluations^45^, making it particularly suitable for this task. We prompted Llama-3-70B to assess all cases with the following instructions:

“*You are a senior radiologist. Below, you will find a case description for a patient diagnosed with [Diagnosis]. Please check if the diagnosis or any part of it is mentioned, discussed, or suggested in the case description. Respond with either ‘mentioned’ (if the diagnosis is included) or ‘not mentioned,’ and nothing else*.”

Subsequently, we prompted each of the sixteen LLMs (15 open-source LLMs + GPT-4o) to provide three differential diagnoses along with a brief rationale for each, using the concatenated “Clinical History” and “Imaging Findings” as input:

“*You are a senior radiologist. Below, you will find information about a patient: first, the clinical presentation, followed by imaging findings. Based on this information, name the three most likely differential diagnoses, with a short rationale for each*.”

Finally, we again utilized Llama-3-70B to evaluate each LLM’s responses on a binary scale, categorizing them as either “correct” (if the correct diagnosis was among the three differential diagnoses) or “wrong.” The prompt for this evaluation was:

“*You are a senior radiologist. Below, you will find the correct diagnosis (indicated after ‘Correct Diagnosis:’) followed by the differential diagnoses provided by a Radiology Assistant during an exam. Please assess whether the Radiology Assistant included the correct diagnosis in their differential diagnosis. Respond only with ‘correct’ (if the correct diagnosis is included) or ‘wrong’ (if it is not)*.”

**Fig. 4:**
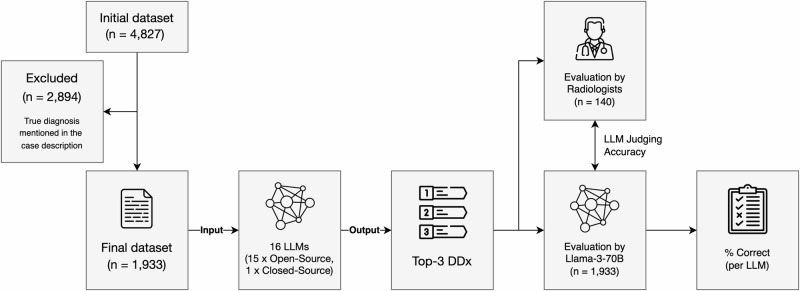
Study design. A total of 2894 cases were excluded as the true diagnosis was mentioned in the case description to be provided as LLM input. 16 LLMs were prompted to output the three most likely differential diagnoses, based on the Eurorad case reports. Llama-3-70B was used to automatically determine the % of correct responses, given the ground truth diagnosis. A subset of 140 LLM responses were additionally rated by radiologists to evaluate the judging accuracy of Llama-3-70B. DDx differential diagnoses.

An exemplary case with details of the LLM query and response evaluation is shown in Fig. 4.

### Human evaluation

**Fig. 5:**
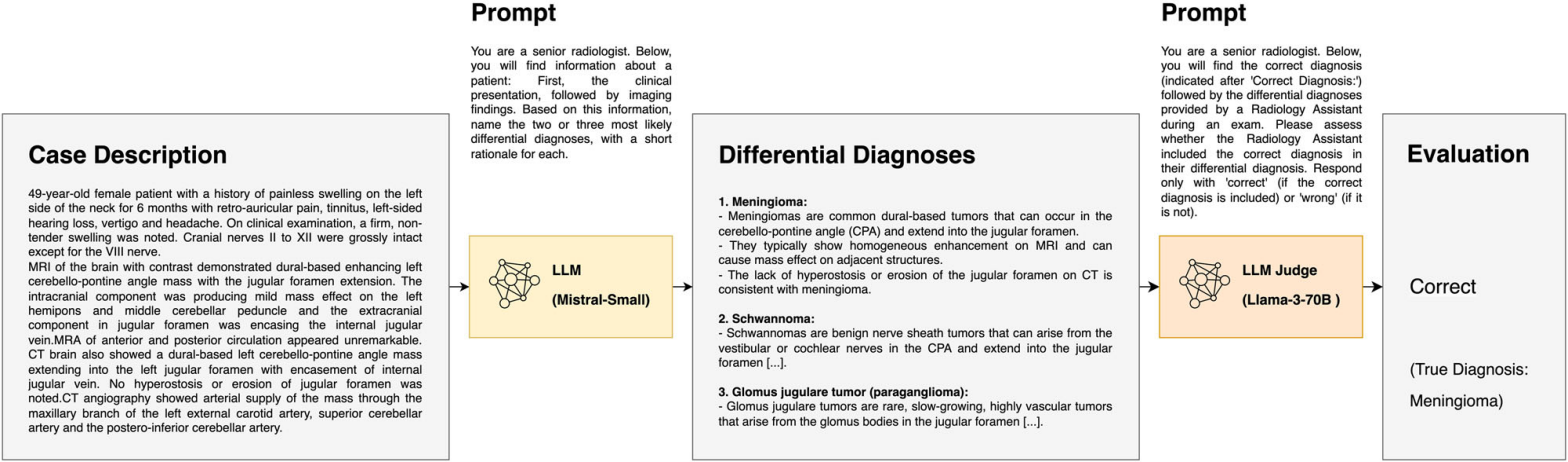
Exemplary LLM query and analysis (Eurorad case ID 12746). Mistral-Small was instructed to determine the most likely differential diagnoses based on a textual case description that included a condensed medical history and imaging findings. The LLM output contained three diagnoses with a rationale for each suggestion. Llama-3-70B was subsequently utilized to classify the response as correct (if the true diagnosis is included in the suggestions) or wrong. The true diagnosis in this case was ‘jugular foramen meningioma’.

In order to gain an understanding of Llama-3-70B’s performance as an LLM judge for correctness of diagnoses, three experienced radiologists (SHK, with 2 years of experience, DMH and BW, board-certified radiologists with 10 years of experience each) additionally evaluated 60 LLM responses each for correctness, of which 20 were shared between all three reviewers to assess human interrater agreement. Using a total of 140 LLM responses for which both human “ground truth” and LLM judge assessments were known, we calculated the accuracy of the LLM judge (Fig. 5).

### Statistics

Both the LLM judge as well as human raters evaluated LLM responses on a binary scale, i.e., if the correct diagnosis was among the top three differential diagnoses listed by the LLM or not. From this response data, we calculated the standard error per model and category as:

1 $$SE=\sqrt{\frac{p(1-p)}{n}}$$

where *p* is the proportion of correct responses, and *n* is the number of samples.

However, from our human evaluation of the LLM judge performance, we know about its inaccuracies and have to adjust the SE to account for this:

2 $$S{E}_{adj}=\sqrt{\frac{A\,\ast \,(1-A)}{n}\,\ast \,{SE}^{2}}$$

where *A* is the accuracy of the LLM judge. The adjusted 95% Confidence Interval is then:

3 $$95{\rm{ \% }}CI=p\pm 1.96\,\ast \,S{E}_{adj}$$

To assess the relationship between LLM size (measured by the number of parameters) and diagnostic accuracy, the Pearson correlation coefficient was calculated.

## Supplementary information

supplement-1

## Data Availability

The Eurorad case report library is publicly accessible at https://eurorad.org/.

## Code availability

Our Python code for prompt construction, along with detailed links to all models (downloaded from https://huggingface.co/), is publicly available in our GitHub repository at https://github.com/ai-idt/os_llm_eurorad.

## References

[1] Gertz, R. J. et al. GPT-4 for automated determination of radiologic study and protocol based on radiology request forms: a feasibility study. *Radiology* **307**, e230877 (2023). 37310247 10.1148/radiol.230877

[2] Rau, A. et al. A context-based chatbot surpasses radiologists and generic ChatGPT in following the ACR appropriateness guidelines. *Radiology* **308**, e230970 (2023). 37489981 10.1148/radiol.230970

[3] Kottlors, J. et al. Feasibility of differential diagnosis based on imaging patterns using a large language model. *Radiology* **308**, e231167 (2023). 37404149 10.1148/radiol.231167

[4] Schramm, S. et al. Impact of Multimodal Prompt Elements on Diagnostic Performance of GPT-4V in Challenging Brain MRI Cases. *Radiology* **314**, e240689 (2025). 39835982 10.1148/radiol.240689

[5] Mallio, C. A., Sertorio, A. C., Bernetti, C. & Beomonte Zobel, B. Large language models for structured reporting in radiology: performance of GPT-4, ChatGPT-3.5, Perplexity and Bing. *Radiol. Med.* **128**, 808–812 (2023). 37248403 10.1007/s11547-023-01651-4

[6] Doshi, R. et al. Quantitative evaluation of large language models to streamline radiology report impressions: a multimodal retrospective analysis. *Radiology* **310**, e231593 (2024). 38530171 10.1148/radiol.231593

[7] Le Guellec, B. et al. Performance of an open-source large language model in extracting information from free-text radiology reports. *Radiol. Artif. Intell.* **6**, 230364 (2024). 10.1148/ryai.230364 PMC11294959 38717292

[8] Lehnen, N. C. et al. Data extraction from free-text reports on mechanical thrombectomy in acute ischemic stroke using ChatGPT: a retrospective analysis. *Radiology* **311**, e232741 (2024). 38625006 10.1148/radiol.232741

[9] Katz, U. et al. GPT versus resident physicians — a benchmark based on official board scores. *NEJM AI* **1**, (2024).

[10] Suh, P. S. et al. Comparing diagnostic accuracy of radiologists versus GPT-4V and gemini pro vision using image inputs from Diagnosis Please cases. *Radiology* **312**, e240273 (2024). 38980179 10.1148/radiol.240273

[11] Sonoda, Y. et al. Diagnostic performances of GPT-4o, Claude 3 –Opus, and Gemini 1.5 Pro in “Diagnosis Please” cases. *Jpn. J. Radiol.* **42**, 1231–1235 (2024). 38954192 10.1007/s11604-024-01619-y PMC11522128

[12] Wu, S. et al. Benchmarking open-source large language models, GPT-4 and claude 2 on multiple-choice questions in nephrology. *NEJM AI* **1** (2024).

[13] Sandmann, S., Riepenhausen, S., Plagwitz, L. & Varghese, J. Systematic analysis of ChatGPT, Google search and Llama 2 for clinical decision support tasks. *Nat. Commun.* **15**, 2050 (2024). 38448475 10.1038/s41467-024-46411-8 PMC10917796

[14] Adams, L. C. et al. Llama 3 challenges proprietary state-of-the-art large language models in radiology board–style examination questions. *Radiology* **312**, e241191 (2024). 39136566 10.1148/radiol.241191

[15] Eurorad. Homepage. https://eurorad.org/ (2024).

[16] Liu, F. et al. Large language models in the clinic: a comprehensive benchmark. Preprint at *medRxiv*10.1101/2024.04.24.24306315 (2024).

[17] Jeong, D. P., Garg, S., Lipton, Z. C. & Oberst, M. Medical adaptation of large language and vision-language models: are we making progress? In *Proc. 2024 Conference on Empirical Methods in Natural Language Processing* 12143-12170 (Association for Computational Linguistics, 2024).

[18] Dorfner, F. J. et al. Biomedical large languages models seem not to be superior to generalist models on unseen medical data. Preprint at arXiv:2408.13833 (2024).

[19] Kim, S. H. et al. Human-AI collaboration in large language model-assisted brain MRI differential diagnosis: a usability study. *European Radiology* (in press). 10.1007/s00330-025-11484-6 PMC12350562 40055233

[20] Siepmann, R. et al. The virtual reference radiologist: comprehensive AI assistance for clinical image reading and interpretation. *Eur. Radiol.* **34**, 6652–6666 (2024). 38627289 10.1007/s00330-024-10727-2 PMC11399201

[21] Dratsch, T. et al. Automation bias in mammography: the impact of artificial intelligence BI-RADS suggestions on reader performance. *Radiology* **307**, e222176 (2023). 10.1148/radiol.222176 37129490

[22] Kim, S. H. et al. Automation bias in AI-assisted detection of cerebral aneurysms on time-of-flight MR-angiography. *La radiologia medica* (in press). 10.1007/s11547-025-01964-6 PMC12008054 39939458

[23] Ke, Y. et al. Mitigating cognitive biases in clinical decision-making through multi-agent conversations using large language models: simulation study. *J. Med. Internet Res.* **26**, e59439 (2024). 39561363 10.2196/59439 PMC11615553

[24] Klang, E. et al. A strategy for cost-effective large language model use at health system-scale. *npj Digit. Med.* **7**, 320 (2024). 39558090 10.1038/s41746-024-01315-1 PMC11574261

[25] Gilbert, S., Harvey, H., Melvin, T., Vollebregt, E. & Wicks, P. Large language model AI chatbots require approval as medical devices. *Nat. Med. 2023 29:10* **29**, 2396–2398 (2023). 10.1038/s41591-023-02412-6 37391665

[26] Zhang, Z. et al. Patients’ perceptions of using artificial intelligence (AI)-based technology to comprehend radiology imaging data. *Health Informatics J.* **27**, 14604582211011215 (2021). 33913359 10.1177/14604582211011215

[27] Golchin, S. & Surdeanu, M. Time travel in LLMs: tracing data contamination in large language models. In *12th International Conference on Learning Representations, ICLR 2024* (2023).

[28] Golchin, S. & Surdeanu, M. Data contamination quiz: a tool to detect and estimate contamination in large language models. Preprint at arXiv:2311.06233 (2023).

[29] Balloccu, S., Schmidtová, P., Lango, M. & Dušek, O. Leak, cheat, repeat: data contamination and evaluation malpractices in closed-source LLMs. In *Proc. 18th Conference European Chapter Association Computational Linguistics* 67–93 (Association for Computational Linguistics, 2024).

[30] Dong, Y. et al. Generalization or memorization: data contamination and trustworthy evaluation for large language models. Preprint at arxiv:2402.15938 (2024).

[31] Gemini models. Gemini API. Google AI for developers. https://ai.google.dev/gemini-api/docs/models/gemini (2024).

[32] Models. Anthropic. https://docs.anthropic.com/en/docs/about-claude/models (2024).

[33] Models. OpenAI API. https://platform.openai.com/docs/models/gp (2024).

[34] Wang, W. et al. CogVLM: visual expert for pretrained language models. Preprint at arXiv:2311.03079 (2023).

[35] Bai, J. et al. Qwen-VL: a versatile vision-language model for understanding, localization, text reading, and beyond. Preprint at arXiv:2308.12966 (2023).

[36] Liu, H., Li, C., Li, Y. & Lee, Y. J. Improved baselines with visual instruction tuning. In *Proceedings of the IEEE/CVF Conference on Computer Vision and Pattern Recognition* (2024).

[37] Mohsan, M. M. et al. Vision transformer and language model based radiology report generation. *IEEE Access* **11**, 1814–1824 (2023).

[38] Tanno, R. et al. Collaboration between clinicians and vision–language models in radiology report generation. *Nat. Med.*10.1038/s41591-024-03302-1 (2024). 10.1038/s41591-024-03302-1 PMC11835717 39511432

[39] Horiuchi, D. et al. Comparing the diagnostic performance of GPT-4-based ChatGPT, GPT-4V-based ChatGPT, and radiologists in challenging neuroradiology cases. *Clin. Neuroradiol.* **34**, 779–787 (2024). 38806794 10.1007/s00062-024-01426-y

[40] Wu, C. et al. Can GPT-4V(ision) serve medical applications? Case studies on GPT-4V for multimodal medical diagnosis. Preprint at arXiv:2310.09909 (2023).

[41] Wu, C., Zhang, X., Zhang, Y., Wang, Y. & Xie, W. Towards generalist foundation model for radiology by leveraging web-scale 2D&3D medical data. Preprint at arXiv:2308.02463 (2023). 10.1038/s41467-025-62385-7 PMC12375113 40849424

[42] Blankemeier, L. et al. Merlin: a vision language foundation model for 3D computed tomography. *Res. Sq.*10.21203/RS.3.RS-4546309/V1 (2024).

[43] Sivarajkumar, S., Kelley, M., Samolyk-Mazzanti, A., Visweswaran, S. & Wang, Y. An empirical evaluation of prompting strategies for large language models in zero-shot clinical natural Language processing: algorithm development and validation study. *JMIR Med. Inform.* **12**, e55318 (2024). 38587879 10.2196/55318 PMC11036183

[44] Zheng, L. et al. Judging LLM-as-a-Judge with MT-bench and Chatbot arena. *Adv. Neural Inf. Process Syst.* **36**, 46595–46623 (2023).

[45] Singh Thakur, A., Choudhary, K., Srinik Ramayapally, V., Vaidyanathan, S. & Hupkes Meta, D. Judging the judges: evaluating alignment and vulnerabilities in LLMs-as-judges. Preprint at arXiv: 2406.12624 (2024).

