# Supplementary material for: Benchmarking the diagnostic performance of open source LLMs in 1933 Eurorad case reports
Source: NPJ Digit Med. 2025 Feb 12;8:97. doi: 10.1038/s41746-025-01488-3 (PMC11814077; doi:10.1038/s41746-025-01488-3)
Supplement: Supplementary file 1 — supplement-1 [file 41746_2025_1488_MOESM1_ESM.pdf]

Subspecialty	Model	% Correct	CI_lower	CI_upper
Abdominal imaging	GPT4o	84.0%	78.8%	89.1%
	Meta-Llama-3-70B	77.7%	72.1%	83.2%
	Qwen2.5-32B	70.5%	64.6%	76.4%
	OpenBioLLM-Llama3-70B	68.5%	62.5%	74.4%
	Gemma-2-27B	67.6%	61.6%	73.6%
	Mistral-Small	66.2%	60.2%	72.2%
	Mistral-Nemo	61.6%	55.5%	67.8%
	Mixtral-8x7B	61.6%	55.5%	67.8%
	Meta-Llama-3-8B	60.5%	54.3%	66.6%
	Phi-3-medium-128K	55.9%	49.6%	62.1%
	Meditron-7B	52.4%	46.2%	58.7%
	Llama-2-70B	51.6%	45.3%	57.8%
	BioMistral-7B	51.0%	44.7%	57.3%
	Vicuna-13B	51.0%	44.7%	57.3%
	OpenBioLLM-Llama3-8B	47.6%	41.3%	53.8%
	Medalpaca-13B	40.7%	34.5%	46.9%
Breast imaging	GPT4o	73.8%	60.5%	87.2%
	Meta-Llama-3-70B	70.8%	57.2%	84.4%
	Gemma-2-27B	58.5%	44.1%	72.8%
	Qwen2.5-32B	56.9%	42.5%	71.3%
	Mistral-Small	53.8%	39.4%	68.3%
	OpenBioLLM-Llama3-70B	52.3%	37.8%	66.8%
	Meta-Llama-3-8B	52.3%	37.8%	66.8%
	Phi-3-medium-128K	47.7%	33.2%	62.2%
	Mistral-Nemo	44.6%	30.2%	59.1%
	Llama-2-70B	44.6%	30.2%	59.1%
	Medalpaca-13B	44.6%	30.2%	59.1%
	Mixtral-8x7B	44.6%	30.2%	59.1%
	Vicuna-13B	41.5%	27.2%	55.9%
	Meditron-7B	40.0%	25.7%	54.3%
	BioMistral-7B	36.9%	22.8%	51.1%
	OpenBioLLM-Llama3-8B	36.9%	22.8%	51.1%
Cardiovascular	GPT4o	80.4%	70.8%	89.9%
	Meta-Llama-3-70B	79.5%	69.8%	89.1%
	Mistral-Small	75.0%	65.0%	85.0%
	OpenBioLLM-Llama3-70B	73.2%	63.0%	83.4%
	Mistral-Nemo	70.5%	60.1%	80.9%
	Qwen2.5-32B	69.6%	59.2%	80.1%
	Mixtral-8x7B	67.9%	57.3%	78.4%
	Meta-Llama-3-8B	64.3%	53.5%	75.0%
	Gemma-2-27B	59.8%	48.9%	70.7%

	Llama-2-70B	57.1%	46.2%	68.1%
	Phi-3-medium-128K	56.3%	45.2%	67.3%
	OpenBioLLM-Llama3-8B	54.5%	43.4%	65.5%
	Vicuna-13B	54.5%	43.4%	65.5%
	BioMistral-7B	50.9%	39.8%	62.0%
	Meditron-7B	50.9%	39.8%	62.0%
	Medalpaca-13B	35.7%	25.0%	46.5%
Chest imaging	GPT4o	78.9%	71.5%	86.4%
	Meta-Llama-3-70B	73.2%	65.3%	81.0%
	Qwen2.5-32B	69.5%	61.4%	77.5%
	Gemma-2-27B	64.7%	56.5%	73.0%
	Mistral-Small	63.7%	55.4%	72.0%
	OpenBioLLM-Llama3-70B	63.2%	54.9%	71.4%
	Mixtral-8x7B	60.0%	51.6%	68.4%
	Mistral-Nemo	57.9%	49.5%	66.3%
	Meta-Llama-3-8B	57.9%	49.5%	66.3%
	Phi-3-medium-128K	53.7%	45.2%	62.2%
	OpenBioLLM-Llama3-8B	51.6%	43.1%	60.1%
	Llama-2-70B	51.1%	42.6%	59.5%
	Vicuna-13B	46.3%	37.8%	54.8%
	BioMistral-7B	45.8%	37.3%	54.3%
	Medalpaca-13B	43.7%	35.2%	52.1%
	Meditron-7B	43.7%	35.2%	52.1%
Genital (female) imaging	GPT4o	81.9%	71.1%	92.8%
	Meta-Llama-3-70B	74.7%	63.0%	86.4%
	Mistral-Small	67.5%	55.2%	79.8%
	Gemma-2-27B	65.1%	52.6%	77.5%
	Qwen2.5-32B	65.1%	52.6%	77.5%
	OpenBioLLM-Llama3-70B	60.2%	47.6%	72.9%
	Mixtral-8x7B	57.8%	45.1%	70.6%
	Mistral-Nemo	56.6%	43.9%	69.4%
	Meta-Llama-3-8B	55.4%	42.6%	68.2%
	Phi-3-medium-128K	48.2%	35.3%	61.0%
	Llama-2-70B	47.0%	34.2%	59.8%
	Vicuna-13B	47.0%	34.2%	59.8%
	BioMistral-7B	43.4%	30.6%	56.1%
	OpenBioLLM-Llama3-8B	43.4%	30.6%	56.1%
	Meditron-7B	43.4%	30.6%	56.1%
	Medalpaca-13B	42.2%	29.4%	54.9%
Head & neck imaging	GPT4o	75.8%	66.3%	85.3%
	Meta-Llama-3-70B	70.2%	60.3%	80.1%
	Mistral-Small	62.1%	51.8%	72.4%

	OpenBioLLM-Llama3-70B	62.1%	51.8%	72.4%
	Meta-Llama-3-8B	59.7%	49.3%	70.1%
	Qwen2.5-32B	58.9%	48.5%	69.3%
	Gemma-2-27B	58.9%	48.5%	69.3%
	Mixtral-8x7B	56.5%	46.0%	66.9%
	Mistral-Nemo	53.2%	42.7%	63.7%
	Phi-3-medium-128K	52.4%	41.9%	62.9%
	Llama-2-70B	50.8%	40.3%	61.3%
	Meditron-7B	46.8%	36.3%	57.3%
	BioMistral-7B	46.0%	35.5%	56.5%
	Vicuna-13B	46.0%	35.5%	56.5%
	OpenBioLLM-Llama3-8B	37.9%	27.6%	48.2%
	Medalpaca-13B	25.8%	16.2%	35.4%
Interventional radiology	Meta-Llama-3-70B	85.2%	67.0%	103.4%
	Mistral-Small	81.5%	62.3%	100.6%
	Gemma-2-27B	77.8%	57.8%	97.7%
	GPT4o	77.8%	57.8%	97.7%
	Meta-Llama-3-8B	74.1%	53.5%	94.7%
	OpenBioLLM-Llama3-70B	74.1%	53.5%	94.7%
	Mixtral-8x7B	70.4%	49.2%	91.5%
	Phi-3-medium-128K	70.4%	49.2%	91.5%
	Mistral-Nemo	70.4%	49.2%	91.5%
	Qwen2.5-32B	70.4%	49.2%	91.5%
	Meditron-7B	66.7%	45.0%	88.3%
	Vicuna-13B	63.0%	41.0%	85.0%
	Llama-2-70B	59.3%	37.0%	81.5%
	BioMistral-7B	55.6%	33.1%	78.0%
	OpenBioLLM-Llama3-8B	55.6%	33.1%	78.0%
	Medalpaca-13B	33.3%	11.7%	55.0%
Musculoskeletal system	GPT4o	76.6%	70.4%	82.8%
	Meta-Llama-3-70B	68.8%	62.2%	75.4%
	Gemma-2-27B	59.6%	52.7%	66.5%
	Mistral-Small	58.5%	51.6%	65.4%
	OpenBioLLM-Llama3-70B	56.7%	49.8%	63.7%
	Mixtral-8x7B	54.6%	47.7%	61.6%
	Qwen2.5-32B	54.3%	47.3%	61.2%
	Mistral-Nemo	50.4%	43.4%	57.3%
	Meta-Llama-3-8B	49.6%	42.7%	56.6%
	Phi-3-medium-128K	45.7%	38.8%	52.7%
	Llama-2-70B	43.3%	36.3%	50.2%
	OpenBioLLM-Llama3-8B	41.5%	34.6%	48.4%
	BioMistral-7B	39.7%	32.8%	46.6%

	Vicuna-13B	39.7%	32.8%	46.6%
	Meditron-7B	37.6%	30.8%	44.4%
	Medalpaca-13B	29.8%	23.2%	36.3%
Neuroradiology	GPT4o	79.7%	74.7%	84.7%
	Meta-Llama-3-70B	74.1%	68.8%	79.4%
	Mistral-Small	62.2%	56.6%	67.9%
	Gemma-2-27B	62.0%	56.3%	67.6%
	OpenBioLLM-Llama3-70B	60.5%	54.9%	66.2%
	Qwen2.5-32B	58.4%	52.7%	64.1%
	Mixtral-8x7B	55.2%	49.5%	60.9%
	Mistral-Nemo	54.2%	48.5%	60.0%
	Meta-Llama-3-8B	53.0%	47.3%	58.8%
	Phi-3-medium-128K	51.1%	45.3%	56.8%
	OpenBioLLM-Llama3-8B	45.8%	40.0%	51.5%
	Vicuna-13B	43.8%	38.1%	49.6%
	Meditron-7B	43.1%	37.4%	48.8%
	Llama-2-70B	41.9%	36.2%	47.6%
	BioMistral-7B	41.6%	35.9%	47.3%
	Medalpaca-13B	27.8%	22.5%	33.2%
Paediatric radiology	GPT4o	78.8%	70.1%	87.6%
	Meta-Llama-3-70B	71.5%	62.2%	80.9%
	OpenBioLLM-Llama3-70B	65.7%	56.0%	75.3%
	Mistral-Small	63.5%	53.8%	73.2%
	Qwen2.5-32B	62.0%	52.2%	71.8%
	Gemma-2-27B	61.3%	51.5%	71.1%
	Mixtral-8x7B	59.1%	49.2%	69.0%
	Meta-Llama-3-8B	57.7%	47.7%	67.6%
	Mistral-Nemo	54.7%	44.8%	64.7%
	Phi-3-medium-128K	54.7%	44.8%	64.7%
	Llama-2-70B	48.9%	38.9%	58.9%
	OpenBioLLM-Llama3-8B	41.6%	31.7%	51.5%
	BioMistral-7B	39.4%	29.6%	49.3%
	Vicuna-13B	39.4%	29.6%	49.3%
	Meditron-7B	38.7%	28.9%	48.5%
	Medalpaca-13B	31.4%	21.9%	40.9%
Uroradiology & genital male imaging	GPT4o	80.1%	71.9%	88.4%
	Meta-Llama-3-70B	66.2%	57.1%	75.4%
	Qwen2.5-32B	60.3%	50.9%	69.6%
	Mistral-Small	58.9%	49.5%	68.4%
	Meta-Llama-3-8B	57.0%	47.5%	66.4%
	Gemma-2-27B	57.0%	47.5%	66.4%
	OpenBioLLM-Llama3-70B	57.0%	47.5%	66.4%

	Mixtral-8x7B	55.0%	45.5%	64.5%
	Mistral-Nemo	51.7%	42.1%	61.2%
	Llama-2-70B	49.0%	39.5%	58.5%
	Vicuna-13B	46.4%	36.8%	55.9%
	Phi-3-medium-128K	45.7%	36.2%	55.2%
	BioMistral-7B	45.0%	35.5%	54.5%
	OpenBioLLM-Llama3-8B	44.4%	34.9%	53.9%
	Meditron-7B	38.4%	29.1%	47.8%
	Medalpaca-13B	29.8%	20.8%	38.8%

Supplementary Table 1: Accuracy with 95% confidence intervals by subspecialty and model (Eurorad dataset).
